# Echocardiographic manifestation of hiatus hernia simulating a left atrial mass: Case report

**DOI:** 10.1186/1476-7120-6-46

**Published:** 2008-09-15

**Authors:** Konstantinos C Koskinas, Kostas Oikonomou, Eleni Karapatsoudi, Pantelis Makridis

**Affiliations:** 1 Cardiology Department, General Hospital of Edessa, 58200 Edessa, Greece; 2 1st Cardiology Department, AHEPA University Hospital, Aristotle University of Thessaloniki, 1 St Kyriakidi Street, 54636 Thessaloniki, Greece

## Abstract

**Background:**

Despite the high prevalence of hiatus hernia, a relatively small number of echocardiographically manifested cases have been reported.

**Case Presentation:**

An 82-year old woman presented with acute retrosternal pain indicative of cardiac etiology. Physical examination and biochemical tests, as well as 12-lead electrocardiogram, were normal. A two-dimensional transthoracic echocardiogram was performed and revealed a structure that was considered to represent a left atrial mass. A subsequent computed tomography scan visualized a hiatus hernia in the posterior mediastinum, impinging on the posterior left atrial wall. The intrathoracic displacement of a large part of the stomach was further confirmed by an upper gastrointestinal barium examination.

**Conclusion:**

Hiatus hernia can present as acute chest pain, while its echocardiographic manifestation may resemble a left atrial space-occupying structure. Physicians should be aware of the clinical and sonographic findings to facilitate the differential diagnosis from similarly presenting cardiac entities.

## Background

Hiatus hernia is a frequent entity, characterized by the displacement of the gastroesophageal junction and part of the stomach into the mediastinum. Although it may produce symptoms suggestive of cardiac etiology, only few cases of its echocardiographic manifestation have been reported. We present the case of a patient with an apparent left atrial mass on transthoracic echocardiography, which was subsequently identified as hiatus hernia.

## Case presentation

An 82-year-old woman presented to the Emergency Department complaining of sudden onset chest pain radiating to the epigastrium at rest, with less than 1 hour of duration. Her medical history included chronic heart failure and she was therefore treated with digitalis 0,25 mg/day. On initial examination her blood pressure was 130/80 mmHg. A grade 3/6 holosystolic murmur was audible at the apex. Her lungs were clear to percussion and auscultation. The 12-lead electrocardiogram (ECG) demonstrated sinus rhythm with non-specific "scooping" ST-segment depression in leads III, aVF, V4–V6, attributable to her current medication. Laboratory tests, including cardiac enzymes and cardiac troponin-I, were within normal reference values. The patient was admitted to the Cardiology Department for further investigation.

**Figure 1:**
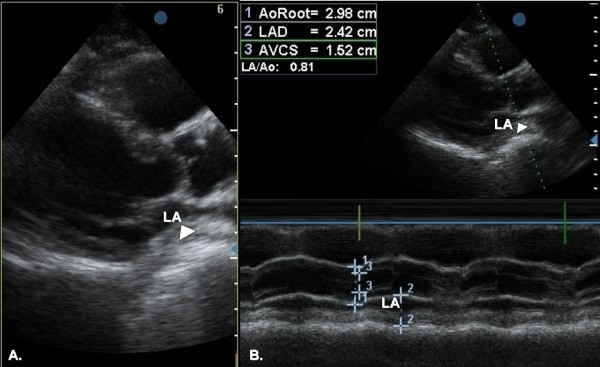
**Two dimensional transthoracic echocardiogram**. A. Echocardiogram in parasternal long-axis shows an echolucent, apparent left atrial (LA) mass (arrowhead). B. M-mode scan through the ascending aorta and left atrium demonstrates the left atrial cavity significantly confined by the apparent mass (arrowhead).

**Figure 2:**
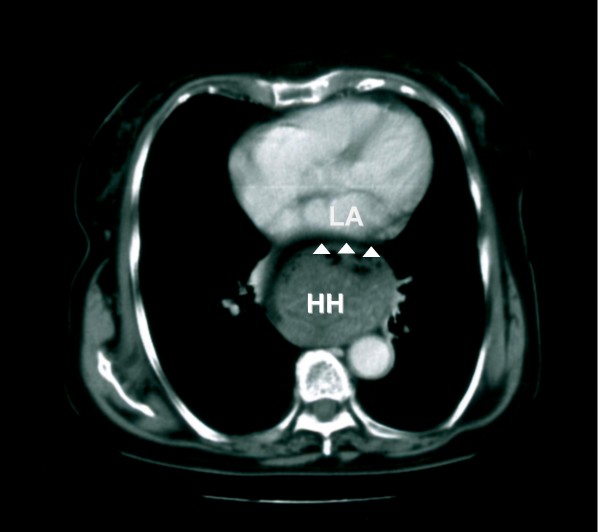
**Thoracic computer tomography scan**. A sizable hiatus hernia (HH) is demonstrated in the mediastinum, impinging on the posterior aspect of the left atrium (LA) (arrowheads).

**Figure 3:**
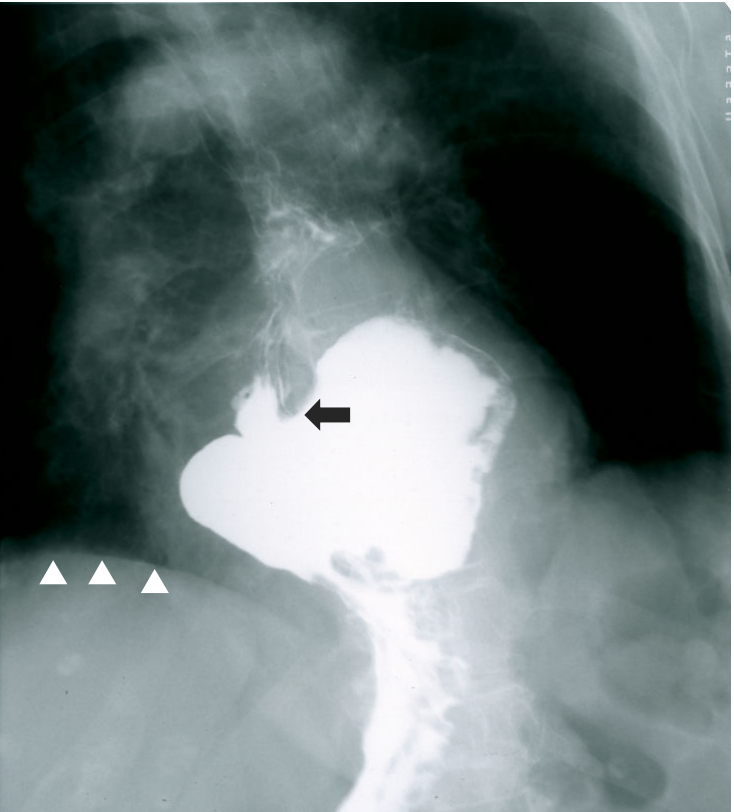
**Upper gastrointestinal barium examination**. The intrathoracic migration of the gastroesophageal junction (arrow) and large part of the stomach above the diaphragm (arrowheads) is confirmed.

A two-dimensional transthoracic echocardiogram, using all standard and modified apical and parasternal views, revealed an amorphous, echolucent mass with the appearance of a left atrial space-occupying lesion (Figure 1, see Additional file 1). Left ventricular contraction was normal and no pericardial effusion was present. The patient subsequently underwent a chest computed tomography (CT) scan; a large hiatus hernia was visualized in the posterior mediastinum (Figure 2). The intrathoracic migration of a large part of the stomach was confirmed by an upper gastrointestinal barium examination, which was performed after consulting a surgeon, to further assess the extent of the hernia and the potential need for surgical treatment (Figure 3).

After excluding the cardiac origin of the clinical presentation, based on serial ECG and biochemical findings, conservative treatment was opted and the patient was discharged two days after admission.

Written consent was obtained from the patient for publication of study.

## Discussion

Although often asymptomatic, hiatus hernia may present with caustic epigastralgia and regurgitation due to concommitant gastroesophageal reflux. It may also exert a wide spectrum of manifestations mimicking acute cardiovascular events. It has thus been implicated with postprandial syncope [1] and angina-like chest pain [2]. ECG changes may result in the misdiagnosis of myocardial ischemia [3]. The clinical presentation of hiatus hernia-induced cardiac compression can range from dyspnea to impaired respiratory function [4], recurrent acute heart failure [5] and ultimately hemodynamic collapse [6]. Further cardiac complications include the formation of gastropericardial fistula [7], pericardial effusion [3] and arrhythmia [2]. Hiatus hernia can hinder the sonographic depiction of the cardiac anatomy [8] and has been reported to simulate the appearance of an intra-atrial mass [9-12] or a posterior mediastinal structure on transthoracic echocardiography [1,3,5].

The intravenous infusion of echocardiographic contrast medium may facilitate the characterization of the investigated structure, according to the degree of enhancement (parallel to the vascular density) and the communication with cardiac chambers [13]. The visualization of swirling echodensities following the oral ingestion of carbonated beverage [14], particularly in combination with echocardiographic contrast media [13], furhter enhances the differential diagnosis. These techniques were regrettably not employed in the present case, inevitably at the expense of the cost-effectiveness of the diagnostic approach, radiation exposure and patient inconvenience.

Various intracardiac or extrinsic lesions can resemble the echocardiographic appearance of hiatus hernia. These include vascular formations, such as descending aorta aneurysm or dilation of the coronary sinus, and inflammatory conditions, as in the case of a mitral ring abscess. Myxoma represents the most frequent primary cardiac tumor. Secondary tumors can infiltrate the cardiac wall per continuitatem, or constitute hematogenous metastases. The augmentation of the mass depiction on posterior imaging planes, the disparate degree of encroachment on the left atrium attributable to respiratory motion [14] and the identification of an inner lining reminiscent of gastric mucosa [15] may distinguish hiatus hernia from similarly presenting structures.

## Conclusion

Hiatus hernia can simulate clinical and sonographic characteristics of cardiac disorders. Its echocardiographic manifestation may mimic a left atrial space-occupying structure; it therefore merits attention for the differentiation from such lesions. Although the definite diagnosis is usually confirmed by other imaging modalities, adequate data can be derived from the appropriate acquisition and interpretation of echocardiographic findings.

## Consent

Written informed consent was obtained from the patient for publication of this case report and any accompanying images. A copy of the written consent is available for review by the Editor-in-Chief of this journal.

## Competing interests

The authors declare that they have no competing interests.

## Authors' contributions

KCK conceived the case report, collected the data, reviewed literature and wrote the manuscript. KO revised the article for important intellectual content. EK participated in the analysis and interpretation of data. PM performed the echocardiogram. All authors read and approved the final manuscript.

## Supplementary Material

Additional file 1 Movie of transthoracic echocardiography. This movie shows a parasternal long-axis echocardiographic view during one cardic cycle demonstrating an apparent left atrial mass. Click here for file
